# *BRCA1* and *BRCA2* mutations in ovarian cancer patients from Belarus: update

**DOI:** 10.1186/s13053-021-00169-y

**Published:** 2021-01-21

**Authors:** Alena Savanevich, Olgierd Ashuryk, Cezary Cybulski, Jan Lubiński, Jacek Gronwald

**Affiliations:** 1 Department of Obstetrics and Gynecology, Grodno State Medical University, Grodno, Belarus; 2 International Hereditary Cancer Center, Department of Genetics and Pathology, Pomeranian Medical University, Szczecin, Poland

**Keywords:** *BRCA1* and *BRCA2* mutation, Mutation, Epithelial ovarian cancer, Belarus

## Abstract

**Background:**

Mutations in *BRCA1* and *BRCA2* are well-established risk factors for breast and ovarian cancer. In Central-Eastern European counties, the founder mutations in the *BRCA1* are responsible for a significant proportion of ovarian cancer cases, however, regional differences in the frequencies of various mutations may exist. The spectrum and frequency of *BRCA1/2* mutations between ovarian cancer patients have not yet been precisely established in Belarus.

**Methods:**

Two hundred fourteen consecutive unselected cases of ovarian cancer patients from the region of West Belarus were examined. We studied 13 founder mutations in *BRCA1* (c.5266dupC, c.4035delA, c.5251C > T, c.181 T > G, c.676delT, c.68_69delAG, c.3700_3704delGTAAA, c.1687C > T, c.3756_3759delGTCT) and in *BRCA2* (c.658_659delGT, c.7913_7917delTTCCT, c.3847_3848delGT, c.5946delT) characteristic for Central European population.

**Results:**

A *BRCA1* or *BRCA2* founder mutations were detected in 54 of the 214 (25.2%) ovarian cancer cases. The *BRCA1* c.5266dupC mutation was detected in 28 patients, followed by c.4035delA mutation observed in 18 patients. *BRCA1* c.3756_3759delGTCT, c.68_69delAG, and c.1687C > T were found in 3, 2, and 1 women, respectively. *BRCA2* c.658_659delGT mutation was detected in 2 ovarian cancer patients. The median age of diagnosis of the 54 hereditary ovarian cancers was 57.5 years.

**Conclusions:**

The frequency of 13 causative *BRCA1* and *BRCA2* founder mutations in West Belarus was higher than in other Slavic countries. Testing of *BRCA1* (c.5266dupC, c.4035delA, c.3756_3759delGTCT, c.68_69delAG, c.1687C > T as well as c.181 T > G) and *BRCA2* (c.658_659delGT) mutations should be considered an inexpensive and sensitive test panel for this population.

## Background

Ovarian cancer is the most aggressive gynecological tumor. Because of the deficiency in early detection procedures and the rapid progression of the disease, more than 70% of the ovarian cancer patients are only diagnosed at an advanced stage. The 5-year survival rate remains under 40% despite the advances in treatment over the past decades [1].

In 2018, the age-standardized rate of ovarian cancer per 100,000 cases was 15.4 in Belarus. It was the 3rd highest in the world [2]. An effective strategy allowing for a partial reduction of the incidence is to identify carriers of mutations predisposing to ovarian cancer and to apply appropriate prophylactic measures in this group.

It has been shown that ovarian cancer patients from Belarus are characterized by a high proportion of a limited number of recurrent mutations in the *BRCA1* gene. Two *BRCA1* founder mutations (c.5266dupC, and c.4035delA) account for approximately 80% of all detectable *BRCA1* and *BRCA2* mutations in breast-ovarian cancer families [3–6]. It was observed that more than 16% of ovarian cancer patients are carriers of these mutations. A high proportion of *BRCA*-carriers with a limited number of recurrent mutations have an impact on the test costs reduction. It also enhances the availability and effectiveness of testing and implementation of effective prophylactics. Recently, several other less frequent recurrent *BRCA1* and *BRCA2* mutations have been reported as well as some regional differences in mutation frequency and spectrum have been observed in central European counties [7–12]. This study aimed to evaluate the prevalence and spectrum of 13 *BRCA1* and *BRCA2* founder mutations most frequently observed in the area of Central Europe in unselected patients from Belarus diagnosed with ovarian cancer. These observations may be important to optimize the genetic testing strategy for ovarian cancer patients in this country.

## Methods

Ovarian cancer cases were identified among patients treated at the clinical base of the Grodno State Medical University in Grodno University Clinic, between June 2016 and July 2019. All patients were inhabitants of the western region of Belarus. The study group consisted of 214 consecutive, newly diagnosed cases of epithelial ovarian cancer after surgical treatment, unselected for age, or family history. This group constituted 83% of the total number of patients with epithelial ovarian cancer who underwent surgery in this period in Grodno University Clinic. The mean age of diagnosis was 58.2 ± 15.2 years (range 27–83 years). The reference pathologist reviewed a representative slide from each cancer to confirm the diagnosis. The cancer family history was obtained through a questionnaire. Each patient provided written informed consent to take part in the study. The study was approved by the ethics committee of Pomeranian Medical University.

DNA was isolated from 5 to 10 ml of blood. All women were tested for the presence of 13 founder mutations in *BRCA1* (nine mutations) and *BRCA2* (four mutations). The c.4035delA and c.5266dupC mutations were detected using allele-specific oligonucleotide PCR. The other mutations of *BRCA1* (c.5251C > T, c.181 T > G, c.676delT, c.68_69delAG, c.3700_3704delGTAAA, c.1687C > T, c.3756_3759delGTCT) and in BRCA2 (c.658_659delGT, c.7913_7917delTTCCT, c.3847_3848delGT, c.5946delT) were genotyped using TaqMan assays (Applied Biosystems/Life Technologies, Carlsbad, CA) on Roche LightCycler 480. All mutations were confirmed by Sanger direct sequencing. Sequencing reactions were performed using a BigDye Terminator v3.1 Cycle Sequencing Kit (Life Technologies) according to the manufacturer’s protocol. Sequencing products were analyzed on the ABI Prism 3100 Genetic Analyzer (Life Technologies) [8].

### Statistical analysis

Categorical data was summarized using percentages; numerical data was summarized as means and standard deviations or medians and ranges. Percentages were statistically compared by the dint of Boschloo test, implemented at the package “Exact” of an extension of the “R” programming language. All tests of hypotheses were conducted at an alpha level of 0.05.

## Results

A *BRCA1* or *BRCA2* gene mutation was detected in 54 of the 214 (25.2%) women affected with epithelial ovarian cancer. Among these the most frequently detected mutations were c.5266dupC and c.4035delA in *BRCA1* gene detected in 46 (21.5%) women, however, as many as 8 (3.7%) patients were carriers of other *BRCA1* or *BRCA2* mutations observed in neighboring Central European countries (Table 1).

**Table 1:** The frequency of *BRCA1* and *BRCA2* founder mutations in unselected series of ovarian cancer patients

Gene	Mutation type	Number of carriers	%
*BRCA1*	c.5266dupC	28	13.1
*BRCA1*	c.4035delA	18	8.4
*BRCA1*	c.3756_3759delGTCT	3	1.4
*BRCA1*	c.68_69delAG	2	0.9
*BRCA1*	c.1687C > T	1	0.5
*BRCA2*	c.658_659delGT	2	0.9
Total		54	25.2

The 5382insC *BRCA1* mutation was the most common - it was diagnosed in 28 patients (13.1%), followed by the 4153delA mutation in 18 patients (8.4%). The median age of diagnosis of the 54 hereditary ovarian cancers was 57.5 ± 10.9 years (range 38–78 years), compared with a median age of diagnosis of 58 ± 12.8 years (range 27–83 years) for the 160 cases without a mutation. A mutation was found significantly more frequently in women diagnosed with ovarian cancer at or under the age of 50–35.2% of patients, compared to 21.9% of women diagnosed at a later age (*p* = 0.0464) (Table 2).

**Table 2:** Prevalence of *BRCA1* and *BRCA2* mutations in ovarian cancer by age of onset and family history

	Number of cases	Number with a mutation	Proportion with a mutation (%)	*p*-value
Age group				
-50	54	19	35.2	0.0464
51+	160	35	21.9	
Family history				
Positive for any cancer	115	40	34.8	
Negative for any cancer	96	14	14.6	0.0009
Positive for OC/BC	55	28	50.9	
Negative for OC/BC	156	26	16.7	0.0002
Unknown	3	–	1,4	

Among the 54 women with ovarian cancer and a *BRCA1* mutation, only 28 reported a first- or second-degree relative with breast or ovarian cancer (50.9%). A mutation was present in 16.7% of women with a negative breast/ovarian family history (*p* = 0.0002) (Table 2). A higher frequency of mutation carriers (34.8% vs. 14.6%) was observed in patients with a positive family history, including a history of any cancer (*p* = 0.009) (Table 2). BRCA1 mutation was also detected in 6 of 13 women with previously detected primary breast cancer.

## Discussion

Belarus is the country situated in the center of Europe with a population of about 10 million people and borders Poland, Lithuania, Latvia, Russia, and Ukraine. We found that approximately 25.2% (54 patients) of unselected cases of ovarian cancer carried one of only six founder mutations in the *BRCA1* or *BRCA2* gene. This is the third study of *BRCA1* mutations in ovarian cancer patients from Belarus. In our previous study, we observed *BRCA1* founder mutation only in 16% of ovarian cancer patients [6]. Bogdanova and colleagues reported a prevalence of mutations of 26% for ovarian cancer patients from the region of Minsk [5]. It should be noted that previous studies were based on the detection of three (c.5266dupC, c.4035delA, and c.181 T > G) *BRCA1* founder mutations. It is possible that there are regional differences in the distribution of mutations within the country, however, the relative distribution of the founder mutations in the two regions was roughly similar. In all studies, the *BRCA1* c.5266dupC mutation was the most common, it accounted for 49–60% of all detected mutations [4, 6]. The c.5266dupC mutation is the most common mutation in other Slavic countries such as Poland [8, 12], Russia [10], Czech Republic [13] Slovenia [14], as well as in northern Greece [15] It is the second most common *BRCA1* mutation in the Ashkenazi Jewish population [16, 17].

*BRCA1* c.4035delA mutation was the second most frequent mutation detected in this study, observed in 33% of carriers. Previously it was observed also in high frequency - 32-45% [4, 6].

In this study we have not found *BRCA1* c.181 T > G carriers, however, it was observed in low frequency by Bogdanova et al. and our group in previous studies [4, 6].

Herein, for the first time, among Belarussian ovarian cancer patients, we detected *BRCA1* c.3756_3759delGTCT, c.68_69delAG, c.1687C > T, and *BRCA2* c.658_659delGT mutations. The frequency of these mutations was relatively low (1.8–5.5% of carriers), however, we think that they should be included in the test panel for the Belarussian population. The higher prevalence of *BRCA* mutations observed in Belarus compared to the other regions may be attributed to the low migration rate.

Similarly, to previous research, a family history of breast or ovarian cancer was negative in every second case of mutation-positive patients; however, reported family history was a strong predictor of the presence of the mutation.

The mean age of ovarian cancer diagnosis of the 54 cases with *BRCA1* or *BRCA2* mutations was 57.5 years, which is higher than the mean age observed in other regions of Central Europe [9, 18]. Possibly there are lifestyle/environmental factors that may influence later age of diagnosis, however, for non-carriers, the mean age of diagnosis was similar in Belarus and in other countries.

There are several limitations to our study. We could not obtain a DNA sample for testing from the 17% of patients who had surgery in Grodno University Clinic (due to the patients’ refusal to be tested or patients’ death). We screened only for the thirteen recurrent mutations in *BRCA1* and *BRCA2* genes and, therefore, it is possible that other germline mutations have been missed. Further studies based on the NGS of *BRCA1* and *BRCA2* genes, as well as other genes that may be associated with a strong predisposition to ovarian cancer, should be carried on. Nevertheless, we think that it is inexpensive and extremely efficient to offer to test all breast and ovarian cancer patients in Belarus for the seven *BRCA1* and *BRCA2* founder mutations already detected in this population, especially in the context of the novel possibilities of breast or ovarian cancer treatment with the use individualized treatment with platin agents, Parp inhibitors, or mitomycin C [19, 20].

It should be noted that the incidence of ovarian cancer and the percentage of cases of carrier-induced *BRCA1* or *BRCA2* mutations are among the highest in the world. On the other hand, only two *BRCA1* mutations (c.5266dupC c.4035delA) are detectable in approximately 90% of carriers, which may be inexpensive to test for. It seems that testing could also be carried out in the scale of the entire population, which, with the introduction of appropriate prophylaxis (prophylactic surgery in carrier woman), should significantly reduce the incidence and mortality of ovarian cancer.

## Data Availability

The datasets used and/or analyzed during the current study are available from the corresponding author on reasonable request.

## Abbreviations

PCR: Polymerase chain reaction
OC: Ovarian cancer
BC: Breast cancer
